# Supplementary material for: Peripheral Immune Profiles in Individuals at Genetic Risk of Amyotrophic Lateral Sclerosis and Alzheimer’s Disease
Source: Cells. 2025 Feb 10;14(4):250. doi: 10.3390/cells14040250 (PMC11852917; doi:10.3390/cells14040250)

Supplementary Figure S1. Age distribution in the Berlin Aging Study II

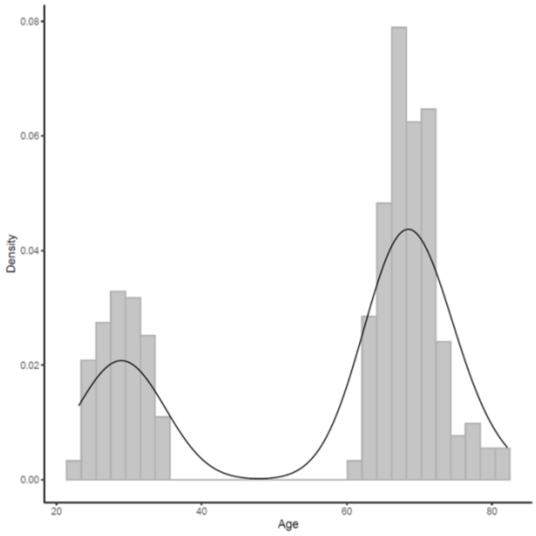

Supplementary Figure S2. Distributions of the polygenic risk scores for amyotrophic lateral sclerosis (A) and Alzheimer's disease (B)

2A.

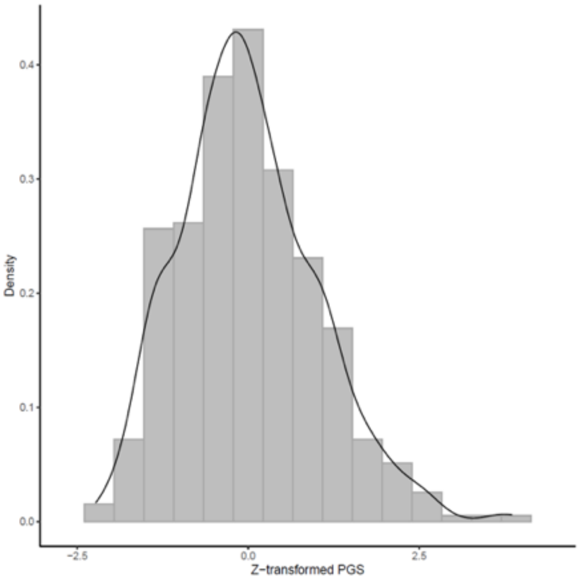

2B.

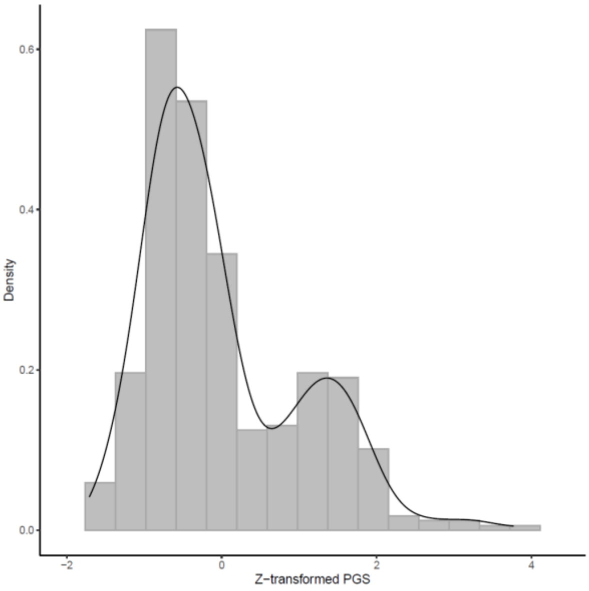

Supplementary Figure S3. Effect size estimates of the polygenic risk scores for amyotrophic lateral sclerosis (ALS) and for Alzheimer’s disease (AD) on all immune cell distributions analyzed

3A. ALS, all participants

Immune cell type	Beta	P value
M_N	-0.141	0.015
CD8+ME_CD57+	-0.134	0.005
CD8+PD-1+	-0.111	0.024
mDC	-0.108	0.047
CD4+ME_CD57+	-0.106	0.038
CD4+M_CD28+	-0.089	0.076
CD4+M_CD27+	-0.085	0.099
CD15high	-0.082	0.138
CD4+ME_CD95+	-0.072	0.150
Lin-HLA-DR-cross	-0.071	0.186
CD4+CM_EM	-0.069	0.155
CD3-CD16-	-0.063	0.253
CD4+LEM	-0.062	0.211
CD8+LD_CD27+	-0.061	0.236
Lin-HLA-DR-	-0.059	0.262
CD14-CD15-	-0.059	0.265
CD8+naive_CD57+	-0.055	0.282
CD4+naive_CD57+	-0.052	0.307
CD8+M_CD27+	-0.049	0.347
CD8+ME	-0.046	0.350
MDSC3	-0.045	0.434
CD8+CD28+	-0.044	0.351
CD8+CD57+	-0.043	0.357
CD8+ME_CD95+	-0.043	0.364
MDSC4	-0.043	0.430
CD4+LEM_CD57+	-0.041	0.413
CD4/CD8_Ratio	-0.038	0.430
MDSC1	-0.038	0.480
DC	-0.037	0.519
CD8+CM_EM	-0.036	0.446
CD4+CD57+	-0.032	0.513
CD4+CD27+	-0.030	0.532
Bcells_NKG2A+	-0.027	0.634
CD4+CD28+	-0.026	0.591
MDSC2_w/oCD11b	-0.025	0.656
Tcells_NKG2A+	-0.024	0.636
CD4+ED_CD27+CD28-	-0.019	0.711
MDSC2	-0.018	0.745
CD8+ED_CD27-CD28-	-0.018	0.725
NKT_CD16+	-0.016	0.781
CD4+ME	-0.011	0.825
CD8+TEMRA	-0.011	0.824
CD8+ED_CD27+CD28-	-0.009	0.854
CD4+LD_CD27+	-0.008	0.868
Lymphocytes_living	-0.006	0.904
CD4+	-0.006	0.909
CD4+ED_CD27-CD28-	-0.005	0.928
CD8+M_CD28+	-0.002	0.966
CD8+CD27+	0.000	0.996
CD14+CD15-	0.001	0.985
CD8+LEM_CD57+	0.002	0.967
Monocytes_CD14+	0.003	0.962
Tcells	0.004	0.934
NKT_CD3+	0.005	0.932
NKT_CD56+	0.006	0.919
NK_mature_NKG2C+	0.006	0.917
Lymphocytes	0.010	0.858
CD4+TEMRA	0.011	0.822
CD4+SM	0.012	0.823
MDSC_CD14+CD15+	0.012	0.830
Monocytes	0.014	0.807
CD8+LEM	0.014	0.787
CD8+LD_CD28+	0.015	0.731
NK_mature_NKG2A+	0.016	0.762
Tcells_NKG2C+	0.020	0.729
Bcells_NKG2C+	0.022	0.692
CD3bright	0.022	0.690
CD4+ED_CD27-CD28+	0.026	0.623
M_C	0.028	0.645
NK_mature	0.028	0.601
CD8+LD	0.028	0.557
CD4+LD_CD28+	0.029	0.576
CD8+ED	0.030	0.450
CD8+naive	0.033	0.402
CD3-CD16+	0.035	0.525
Bcells	0.036	0.519
CD8+SM	0.036	0.470
sDC	0.037	0.545
CD8+LD_CD27+CD28+	0.039	0.341
Tcells_leuko	0.039	0.452
CD8+ED_CD27-CD28+	0.040	0.428
M_I	0.046	0.427
CD8+	0.049	0.310
CD4+naive	0.055	0.253
CD4+LD	0.055	0.262
NK_immature_NKG2C+	0.060	0.289
CD4+ED	0.060	0.212
CD4+LD_CD27+CD28+	0.064	0.180
NK	0.065	0.243
Lin-CD14+HLA-DR+	0.072	0.194
NK_immature_NKG2A+	0.076	0.175
NK_immature	0.079	0.157

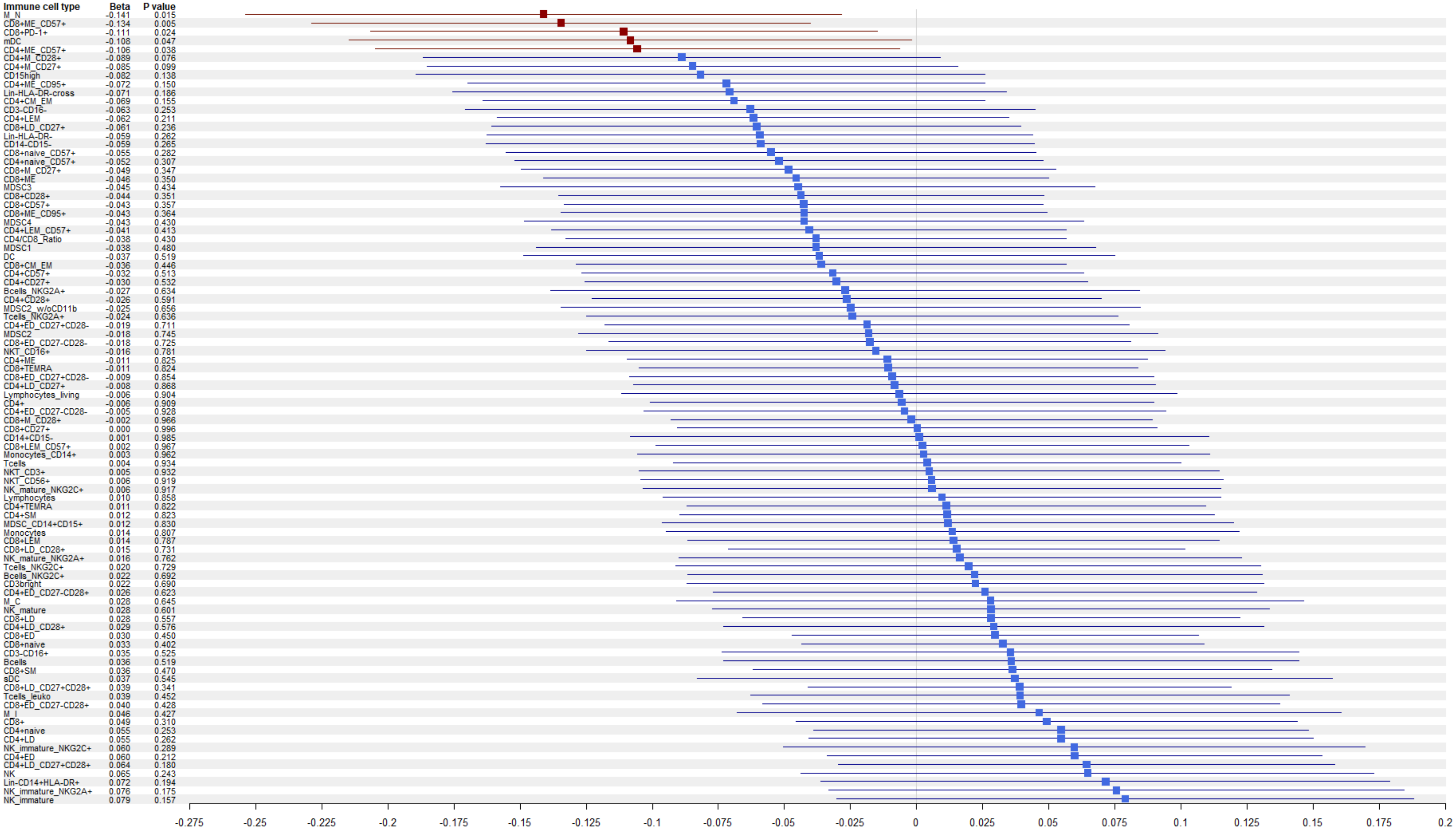

3B. ALS, older age group

Immune cell type	Beta	P value
CD8+ME_CD57+	-0.155	0.012
CD4+ME_CD57+	-0.118	0.057
M_N	-0.117	0.129
CD4+M_CD27+	-0.097	0.118
CD8+PD-1+	-0.095	0.116
CD4+M_CD28+	-0.087	0.160
NKT_CD16+	-0.085	0.213
CD8+naive_CD57+	-0.082	0.188
CD4+naive_CD57+	-0.074	0.236
CD8+LD_CD27+	-0.071	0.258
CD15high	-0.069	0.313
CD8+M_CD27+	-0.062	0.320
DC	-0.055	0.452
CD8+CD28+	-0.052	0.389
CD8+CD57+	-0.052	0.381
CD4+LEM	-0.048	0.433
CD4/CD8_Ratio	-0.047	0.446
MDSC2_w/oCD11b	-0.047	0.492
CD8+ME	-0.046	0.449
CD3-CD16-	-0.046	0.502
MDSC3	-0.045	0.525
mDC	-0.045	0.507
CD8+ME_CD95+	-0.044	0.472
CD4+CM_EM	-0.043	0.483
CD8+TEMRA	-0.042	0.502
CD14-CD15-	-0.041	0.547
MDSC2	-0.041	0.548
CD14+CD15-	-0.041	0.553
CD4+ME_CD95+	-0.039	0.536
CD4+SM	-0.039	0.545
CD4+LEM_CD57+	-0.039	0.533
CD8+CM_EM	-0.034	0.578
CD4+ED_CD27+CD28-	-0.031	0.616
CD8+ED_CD27-CD28-	-0.029	0.633
Lin-HLA-DR-cross	-0.026	0.702
CD8+LD_CD28+	-0.024	0.693
Lin-HLA-DR-	-0.020	0.771
CD4+ED_CD27-CD28+	-0.019	0.766
CD8+CD27+	-0.018	0.766
CD4+	-0.018	0.776
MDSC4	-0.012	0.856
CD4+CD57+	-0.012	0.850
CD8+M_CD28+	-0.012	0.851
Bcells_NKG2A+	-0.010	0.887
NK_mature_NKG2A+	-0.010	0.887
CD4+LD_CD27+	-0.009	0.882
CD4+CD27+	-0.007	0.906
CD4+LD_CD28+	-0.006	0.923
CD8+ED_CD27+CD28-	-0.005	0.934
CD4+CD28+	-0.004	0.942
CD3-CD16+	-0.004	0.958
CD8+SM	0.000	0.994
Monocytes	0.001	0.989
CD4+ED_CD27-CD28-	0.003	0.961
Tcells_NKG2A+	0.005	0.941
Monocytes_CD14+	0.007	0.922
MDSC1	0.007	0.918
CD8+ED_CD27-CD28+	0.009	0.886
CD8+LEM_CD57+	0.011	0.859
NK_mature_NKG2C+	0.017	0.798
CD8+LEM	0.023	0.720
CD8+LD	0.024	0.708
NK_mature	0.025	0.715
CD4+naive	0.025	0.678
Lymphocytes_living	0.026	0.706
Tcells	0.026	0.680
NKT_CD3+	0.028	0.687
NKT_CD56+	0.030	0.667
NK	0.031	0.663
CD4+TEMRA	0.031	0.613
MDSC_CD14+CD15+	0.032	0.635
CD4+ED	0.033	0.588
CD4+ME	0.035	0.586
CD4+LD_CD27+CD28+	0.035	0.569
CD3bright	0.037	0.585
CD4+LD	0.038	0.532
M_I	0.040	0.595
CD8+ED	0.040	0.496
CD8+naive	0.041	0.490
Tcells_NKG2C+	0.041	0.545
Lymphocytes	0.048	0.479
CD8+LD_CD27+CD28+	0.050	0.414
M_C	0.052	0.482
CD8+	0.053	0.393
sDC	0.053	0.477
NK_immature	0.067	0.331
NK_immature_NKG2A+	0.067	0.327
Tcells_leuko	0.070	0.302
Bcells_NKG2C+	0.071	0.298
Lin-CD14+HLA-DR+	0.072	0.294
Bcells	0.076	0.252
NK_immature_NKG2C+	0.115	0.096

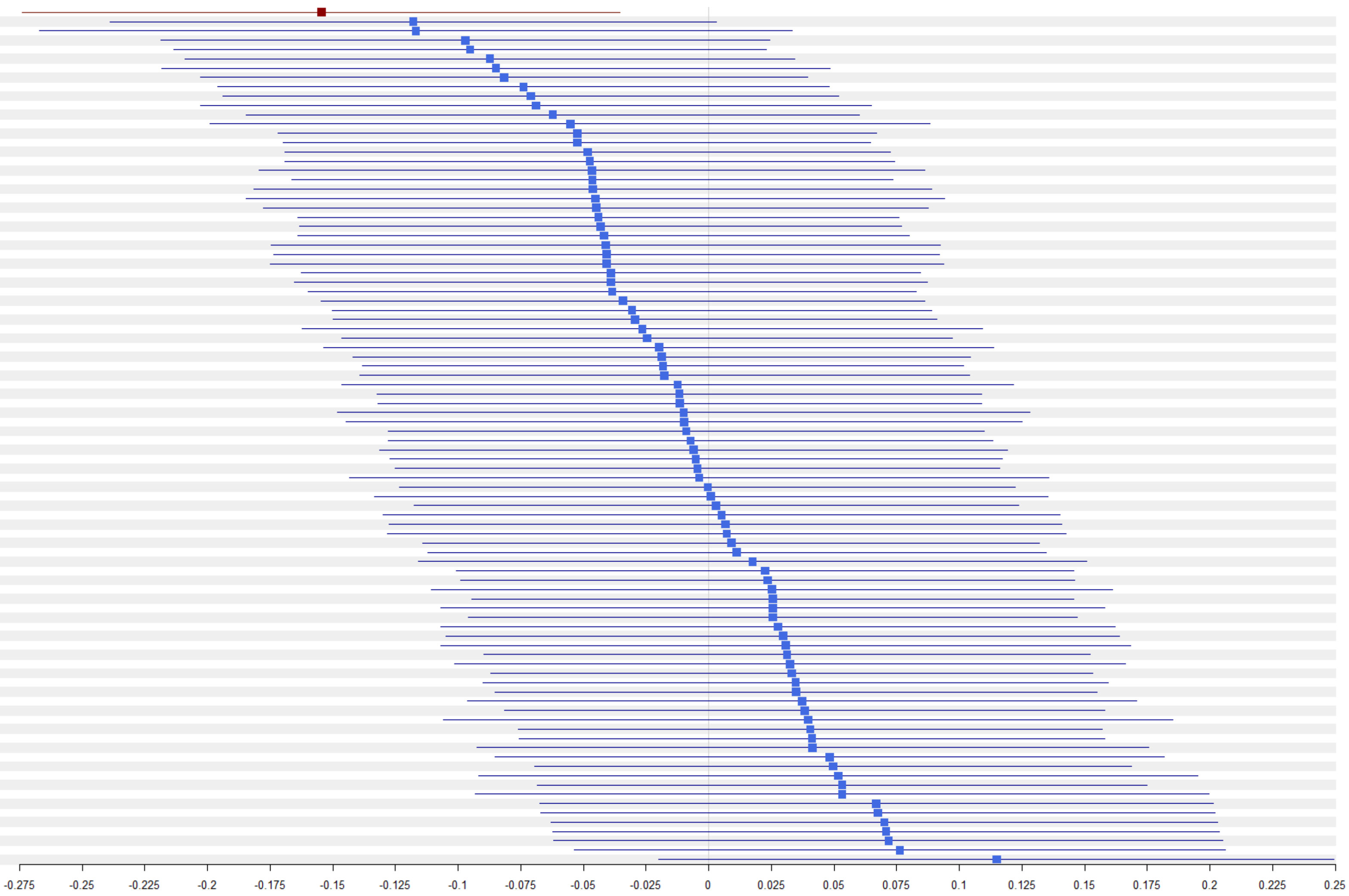

3C. AD, all participants

Immune cell type	Beta	P value
Tcells_NKG2C+	-0.102	0.071
NKT_CD56+	-0.078	0.172
NKT_CD3+	-0.065	0.252
CD8+LEM_CD57+	-0.064	0.217
CD8+LEM	-0.060	0.249
CD4+CD28+	-0.053	0.296
CD4+ED_CD27+CD28-	-0.051	0.322
CD4+SM	-0.051	0.329
Bcells_NKG2C+	-0.051	0.361
CD4+LD_CD27+	-0.046	0.380
CD8+ED_CD27-CD28-	-0.042	0.418
CD8+CD28+	-0.042	0.385
CD8+CM_EM	-0.040	0.407
CD4+ED_CD27-CD28+	-0.036	0.496
CD8+CD27+	-0.032	0.505
CD4+M_CD27+	-0.029	0.577
Tcells	-0.028	0.572
CD4+TEMRA	-0.028	0.586
CD4+ED_CD27-CD28-	-0.026	0.608
CD4/CD8_Ratio	-0.026	0.604
CD8+TEMRA	-0.025	0.606
CD4+ME	-0.025	0.622
Monocytes	-0.020	0.711
CD8+ED_CD27-CD28+	-0.020	0.695
CD3bright	-0.018	0.747
Lymphocytes_living	-0.017	0.756
CD4+LD_CD27+CD28+	-0.014	0.769
CD14+CD15-	-0.012	0.826
Lymphocytes	-0.012	0.820
NK_mature_NKG2C+	-0.010	0.859
CD8+CD57+	-0.009	0.854
sDC	-0.008	0.898
M_N	-0.008	0.895
CD4+ED	-0.006	0.904
CD4+	-0.005	0.925
CD4+CD57+	-0.004	0.928
CD4+LEM	-0.004	0.938
CD4+LD	-0.003	0.955
NK	-0.002	0.968
CD4+naive	-0.002	0.969
CD8+ED_CD27+CD28-	-0.001	0.981
CD4+LEM_CD57+	-0.001	0.982
mDC	0.000	0.993
CD8+PD-1+	0.001	0.980
CD8+ME	0.001	0.979
Tcells_leuko	0.003	0.959
CD8+LD_CD28+	0.003	0.953
M_C	0.003	0.965
CD4+LD_CD28+	0.004	0.943
Bcells	0.004	0.947
CD3-CD16+	0.006	0.916
MDSC2	0.006	0.915
CD8+ME_CD95+	0.007	0.879
MDSC2_w/oCD11b	0.010	0.864
CD4+CD27+	0.012	0.804
M_I	0.015	0.803
CD8+LD	0.015	0.761
NK_mature	0.018	0.745
Monocytes_CD14+	0.018	0.743
Lin-CD14+HLA-DR+	0.020	0.723
CD4+naive_CD57+	0.020	0.698
CD8+ED	0.021	0.600
CD8+M_CD28+	0.023	0.632
CD4+CM_EM	0.023	0.642
CD8+LD_CD27+CD28+	0.028	0.508
CD8+naive	0.028	0.480
CD8+	0.031	0.536
MDSC_CD14+CD15+	0.031	0.574
CD8+LD_CD27+	0.034	0.513
CD4+ME_CD95+	0.034	0.498
CD8+M_CD27+	0.036	0.502
DC	0.039	0.502
MDSC4	0.039	0.470
CD8+ME_CD57+	0.040	0.424
CD3-CD16-	0.041	0.466
Lin-HLA-DR-	0.042	0.432
CD4+ME_CD57+	0.043	0.406
CD8+SM	0.043	0.399
Tcells_NKG2A+	0.046	0.373
NK_immature_NKG2C+	0.047	0.409
CD15high	0.052	0.355
CD4+M_CD28+	0.053	0.299
Lin-HLA-DR-cross	0.054	0.326
CD14-CD15-	0.055	0.302
MDSC1	0.067	0.219
NK_immature	0.071	0.207
Bcells_NKG2A+	0.073	0.208
NK_immature_NKG2A+	0.074	0.189
MDSC3	0.078	0.182
NK_mature_NKG2A+	0.081	0.138
NKT_CD16+	0.081	0.153
CD8+naive_CD57+	0.081	0.118

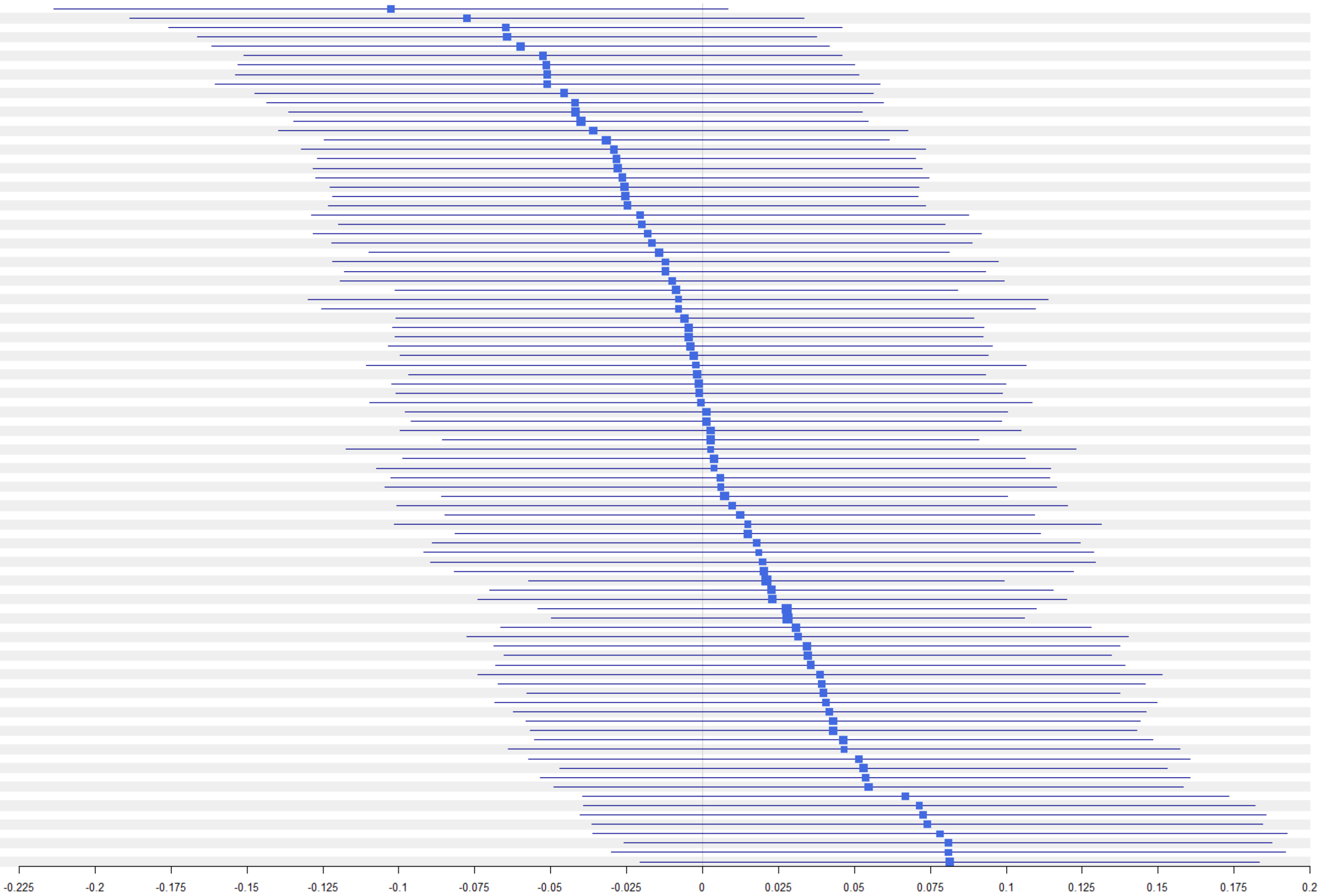

3D. AD, older age group

Immune cell type	Beta	P value
CD8+LEM	-0.133	0.038
NKT_CD3+	-0.123	0.080
CD8+LEM_CD57+	-0.120	0.061
CD8+CM_EM	-0.097	0.127
NKT_CD56+	-0.096	0.170
CD3bright	-0.074	0.288
Tcells_NKG2C+	-0.067	0.340
CD8+CD28+	-0.066	0.297
CD4+ME	-0.066	0.309
CD8+CD27+	-0.059	0.352
CD4+ED_CD27-CD28+	-0.059	0.357
CD8+M_CD28+	-0.048	0.451
CD4+CD27+	-0.047	0.458
CD4+CD28+	-0.043	0.500
Bcells_NKG2C+	-0.043	0.539
M_I	-0.035	0.648
CD4+TEMRA	-0.032	0.613
M_C	-0.030	0.690
CD8+ME	-0.030	0.631
CD4/CD8_Ratio	-0.028	0.664
CD8+TEMRA	-0.026	0.690
Monocytes	-0.020	0.771
Monocytes_CD14+	-0.019	0.789
CD4+SM	-0.018	0.782
MDSC_CD14+CD15+	-0.018	0.798
CD8+ME_CD95+	-0.016	0.800
CD4+CD57+	-0.015	0.808
CD4+M_CD28+	-0.015	0.815
Tcells	-0.015	0.818
CD8+M_CD27+	-0.011	0.869
NK_mature_NKG2C+	-0.009	0.898
CD8+CD57+	-0.008	0.894
CD14+CD15-	-0.008	0.906
CD4+CM_EM	-0.008	0.897
CD8+ED_CD27-CD28-	-0.007	0.909
CD4+LEM	-0.007	0.912
CD4+LD_CD28+	-0.006	0.919
Bcells	-0.006	0.926
mDC	-0.005	0.945
CD8+ED_CD27-CD28+	-0.005	0.942
Lin-CD14+HLA-DR+	-0.003	0.969
CD4+LEM_CD57+	0.000	0.999
CD15high	0.004	0.958
CD4+	0.006	0.925
CD4+ED_CD27-CD28-	0.007	0.906
MDSC4	0.012	0.864
M_N	0.015	0.860
CD4+ME_CD95+	0.016	0.809
MDSC2	0.017	0.807
NK	0.018	0.803
CD8+LD_CD28+	0.018	0.780
CD4+LD_CD27+CD28+	0.020	0.757
MDSC2_w/oCD11b	0.022	0.750
sDC	0.024	0.753
CD3-CD16+	0.026	0.708
CD4+ED	0.027	0.672
NKT_CD16+	0.029	0.683
CD4+naive	0.030	0.629
CD4+LD	0.034	0.595
CD4+LD_CD27+	0.034	0.596
CD8+PD-1+	0.034	0.585
NK_immature_NKG2C+	0.038	0.596
CD8+	0.038	0.551
Tcells_NKG2A+	0.038	0.587
CD4+M_CD27+	0.043	0.504
Tcells_leuko	0.044	0.524
Lymphocytes	0.046	0.506
CD4+ED_CD27+CD28-	0.046	0.463
Lin-HLA-DR-	0.047	0.505
MDSC3	0.051	0.490
CD8+ED_CD27+CD28-	0.057	0.376
Lymphocytes_living	0.059	0.397
CD8+ED	0.059	0.331
DC	0.060	0.421
CD8+naive	0.063	0.307
CD8+LD_CD27+	0.069	0.288
Lin-HLA-DR-cross	0.070	0.330
CD8+LD_CD27+CD28+	0.073	0.242
CD14-CD15-	0.074	0.285
CD3-CD16-	0.074	0.292
CD8+ME_CD57+	0.077	0.231
CD8+SM	0.077	0.230
NK_immature	0.079	0.265
CD8+LD	0.080	0.216
NK_immature_NKG2A+	0.086	0.223
MDSC1	0.087	0.221
NK_mature	0.095	0.181
CD4+ME_CD57+	0.100	0.118
CD4+naive_CD57+	0.102	0.112
Bcells_NKG2A+	0.108	0.139
CD8+naive_CD57+	0.156	0.015
NK_mature_NKG2A+	0.158	0.024

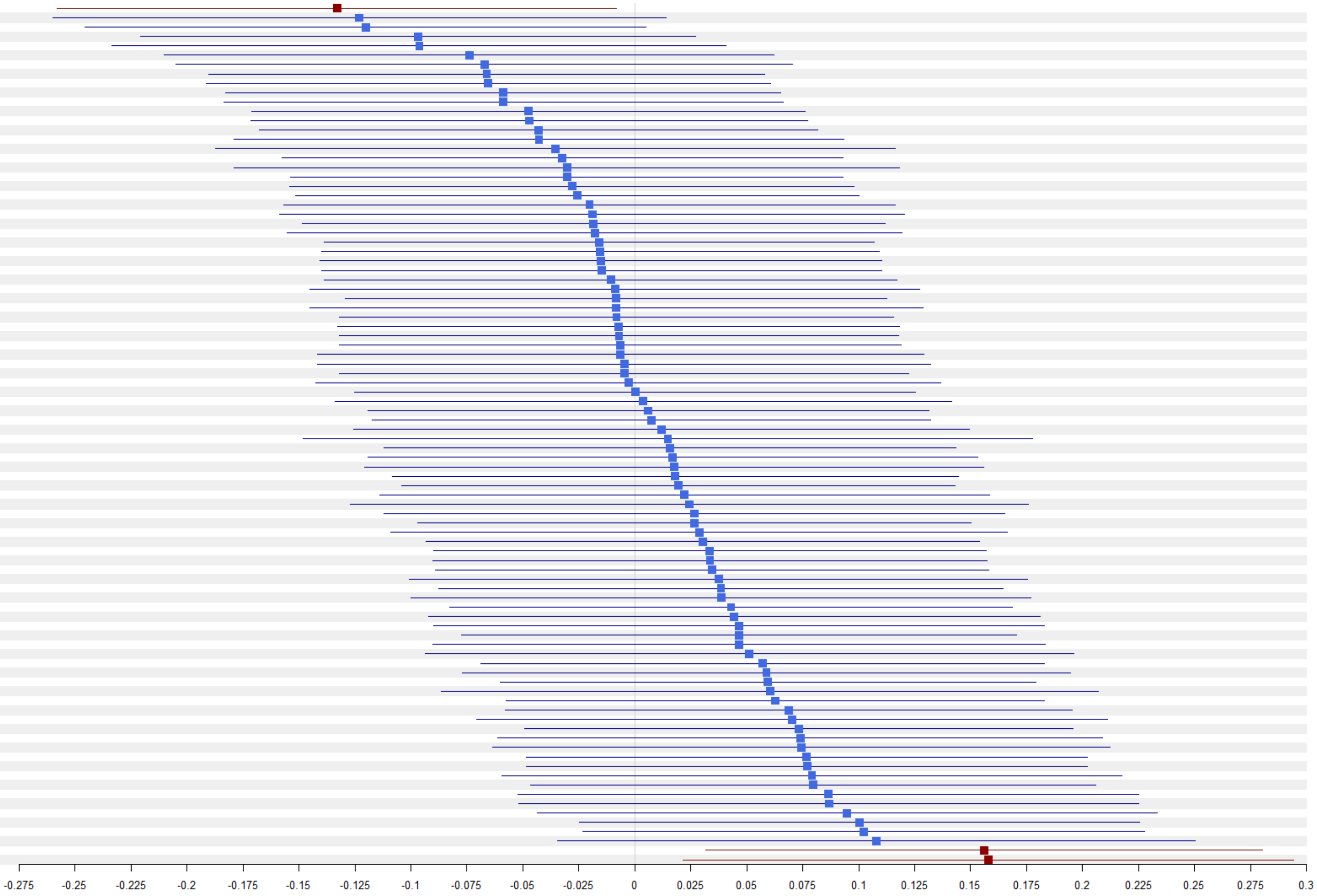

Supplement: Supplementary file 1 [file cells-14-00250-s001.zip › Deecke_immuneAD_ALS_PGS_supplementary_material_final-Cells.pdf]
